# Fetal bovine serum contains biologically available ATP

**DOI:** 10.1007/s11302-023-09941-2

**Published:** 2023-04-19

**Authors:** Valentina Vultaggio-Poma, Leticia Scussel Bergamin, Simonetta Falzoni, Mario Tarantini, Anna Lisa Giuliani, Dorianna Sandonà, Patrizia Polverino De Laureto, Francesco Di Virgilio

**Affiliations:** 1 Department of Medical Sciences, University of Ferrara, Via Borsari 46, 44121 Ferrara, Italy; 2 Department of Biomedical Sciences, University of Padova, Padua, Italy; 3 Department of Pharmaceutical and Pharmacological Sciences, University of Padova, Padua, Italy

**Keywords:** FBS, ATP, Purinergic signaling, P2X7R

## Abstract

ATP is a ubiquitous extracellular messenger released in a wide number of pathophysiological conditions. ATP is known to be present in minute amounts in the extracellular space in healthy tissues and in the blood, and to modulate a multiplicity of cell responses. Cell culture systems are widely used to explore purinergic signaling. We show here that currently used fetal bovine sera contain ATP in the 300–1300 pmol/L range. Serum ATP is associated with albumin as well as with microparticle/microvesicle fraction. Serum microparticles/microvesicles affect in vitro cell responses due to their content of miRNAs, growth factors, and other bioactive molecules. ATP is likely to be one of these bioactive factors found in a variable amount in sera of different commercial sources. ATP in serum supports ATP-dependent biochemical reactions such as the hexokinase-dependent phosphorylation of glucose to glucose 6-phosphate, and affects purinergic signaling. These findings show that cells growing in vitro in serum-supplemented media are exposed to varying levels of extracellular ATP, and thus to varying degrees of purinergic stimulation.

## Introduction

Fetal bovine serum (FBS, also known as fetal calf serum, FCS), sourced from bovine fetuses taken from pregnant cows during slaughter, is the most widely used growth supplement for cell culture media. FBS contains a wide range of nutrients and growth factors as well as undefined components that may unpredictably interfere with in vitro experiments [1]. For instance, it is reported that FBS or components thereof derived, such as bovine serum albumin (BSA), may inhibit formation of neutrophil extracellular traps (NETs) [2], impair cell energy metabolism [3], or heavily influence the analysis of in vitro-generated extracellular vesicles [4]. In fact, FBS contains a variable amount of extracellular vesicles (EVs) or microparticles (MPs) known to carry a multiplicity of bioactive factors [5]. Accordingly, FBS-derived EVs have been shown to affect myoblast proliferation and differentiation [6], to promote cancer cell growth [7], cell migration [8], and adipogenic differentiation of human bone marrow mesenchymal stromal cells [9]. We recently observed that addition of ATP to cell-free, FBS-containing culture medium triggered a transient increase of free ATP (ATP-induced ATP release) [10]. Scattered previous reports showed that BSA binds ATP [11, 12], and that MPs (the rather mixed extracellular vesicle population containing exosomes, canonical EVs and naked mitochondria) are loaded with ATP [10]. Furthermore, as shown by Michel et al. [13] serum components may bind purinergic agonists, i.e., benzoyl ATP, affecting their potency at the P2X7 receptor. Since ATP is the quintessential purinergic agonist we set to identify the main sources of this nucleotide in FBS and to measure its concentration. Our data show that FBS contains variable concentrations of ATP, depending on the commercial source, mainly bound to albumin or associated with the MP fraction. Of note, ATP in FBS is available to support biochemical reactions.

## Materials and methods

### Reagents

Fetal bovine serum (FBS) was purchased from different commercial sources (Sigma-Aldrich, St. Louis, MO, USA, cat#F7524; Gibco, Thermo Fisher Scientific, Waltham, MA, USA, cat#26140079; Euroclone, Milan, Italy, cat#ECS0180L; Microgem, Naples, Italy, cat # RM10432). Bovine serum albumin (BSA, cat # A2153), apyrase from potatoes (cat # A6535), β-nicotinamide adenine dinucleotide^+^ (NAD^+^, cat # N7004), and hexokinase and glucose-6-phosphate dehydrogenase (cat # H8629) were purchased from Sigma-Aldrich.

### In vitro measure of ATP levels

ATP was measured with the ENLITEN rLuciferase/Luciferin reagent (cat # FF2021, Promega, Milan, Italy) with a PerkinElmer Wallac Victor3 1420 luminometer (PerkinElmer, Wellesley, MA, USA). Briefly, 100 µL of FBS (or BSA solution) was placed in 96-well microplates (cat # 655077, Greiner Bio-One Italia, Milan, Italy) and then 2 μL of lysis buffer (not in the case of BSA) and 100 μL of ENLITEN reagent were added to each well. In control experiments, 100 µL of FBS was treated with 1 µg of apyrase for 10 min, before being used for ATP measurement.

### Acid precipitation

One milliliter of FBS (whether from Euroclone, Microgen, Sigma, or Gibco) was denatured with 0.6 N perchloric acid and centrifuged at 4 °C, 16, 400 xg for 20 min. Thereafter, the supernatants were neutralized with 4 N KOH and 6 N of perchloric acid, and centrifuged again at 4 °C and 16,400 × *g* for 20 min. Then, the supernatants were collected for ATP quantification. Briefly, 100 µL of FBS was placed in ninety-six-well microplates and 100 μL of ENLITEN reagent was added to each well.

### Measurement of ATP content of isolated extracellular vesicles/microparticles

Extracellular vesicles/microparticles were purified by FBS centrifugation at 100,000 × *g* in a Beckman L8-M Ultracentrifuge equipped with a 70Ti rotor (Beckman Coulter SpA, Milano, Italy) for 90 min at 4 °C. Microparticle pellets were collected, resuspended in 200 µL of appropriate buffer, and the ATP content measured with the ENLITEN rLuciferase/Luciferin reagent. Luminescence was converted to the ATP concentration with an independent calibration.

### Cytosolic calcium concentration measurement

HEK293 cells stably transfected with the P2X7 receptor (P2X7R) (HEK293-P2X7R) were cultured for 48–72 h in DMEM-F12 (cat # D6421, Sigma-Aldrich) supplemented with 10% of heat FBS from different Gibco, Euroclone, or Sigma. Cytosolic Ca^2+^ was measured with the fluorescent Ca^2+^ indicator Fura-2-acetoxymethyl ester (Fura-2/AM) (cat # F1221, Thermo Fisher Scientific). Briefly, HEK293-P2X7R cells were loaded with 4 µM fura-2/AM, incubated for 30 min at 37 °C, rinsed and resuspended in a saline solution containing 125 mM NaCl, 5 mM KCl, 1 mM MgSO_4_, 1 mM NaH_2_PO_4_, 20 mM HEPES, 5.5 mM glucose, 5 mM NaHCO_3_, 1 mM CaCl_2_ and 250 μM sulfinpyrazone (Sigma), and pH 7.4. Experiments were carried out in a thermostat-controlled (37 °C) and magnetically stirred fluorometer cuvette (LS50, PerkinElmer Ltd. Beaconsfield, UK). [Ca^2+^]_i_ was determined with the 340/380 excitation ratio at an emission wavelength of 500 nm.

### Glucose assay

One hundred microliter of FBS was added to 1 mL of a reagent solution containing 1unit/mL of hexokinase and glucose-6-phosphate dehydrogenase (G6PDH) in the presence of 1.5 mM NAD^+^. Glucose is phosphorylated to glucose-6-phosphate by hexokinase, and glucose-6-phosphate is then oxidized to 6-phosphogluconate by G6PDH in the presence of NAD^+^, which is contextually reduced to NADH. Absorbance increase at 340 nm was measured with a BioSpectrometer spectrophotometer (Eppendorf, Hamburg, Germany).

### Mass spectrometry

Mass spectrometry analysis was carried out with an electrospray ionization (ESI) mass spectrometer equipped with a Q-Tof analyzer Xevo G2S (Waters, Manchester, UK). Measurements were carried out at 2.5 kV capillary voltage and 40 V cone voltage. The source temperature was set at 100 °C and the desolvation temperature at 400 °C. For LC analysis, a C18 column (Acquity UPLC BEH 2.1 × 50 mm, Waters) was used, at room temperature, by using a flow rate of 200 µL/min.

### Statistical analysis

Data were analyzed with the GraphPad Prism 9 software (GraphPad Software, Inc., La Jolla, CA, USA). Statistical significance was calculated with a two-tailed Student’s *t* test assuming equal SD and variance. All data are shown as mean ± standard error of the mean (S.E.M.). Differences were considered significant at *p* < 0.05. Coding: *p ≤ 0.05; **p ≤ 0.01; ***p ≤ 0.001; ****p ≤ 0.0001.

## Results

**Fig. 1:**
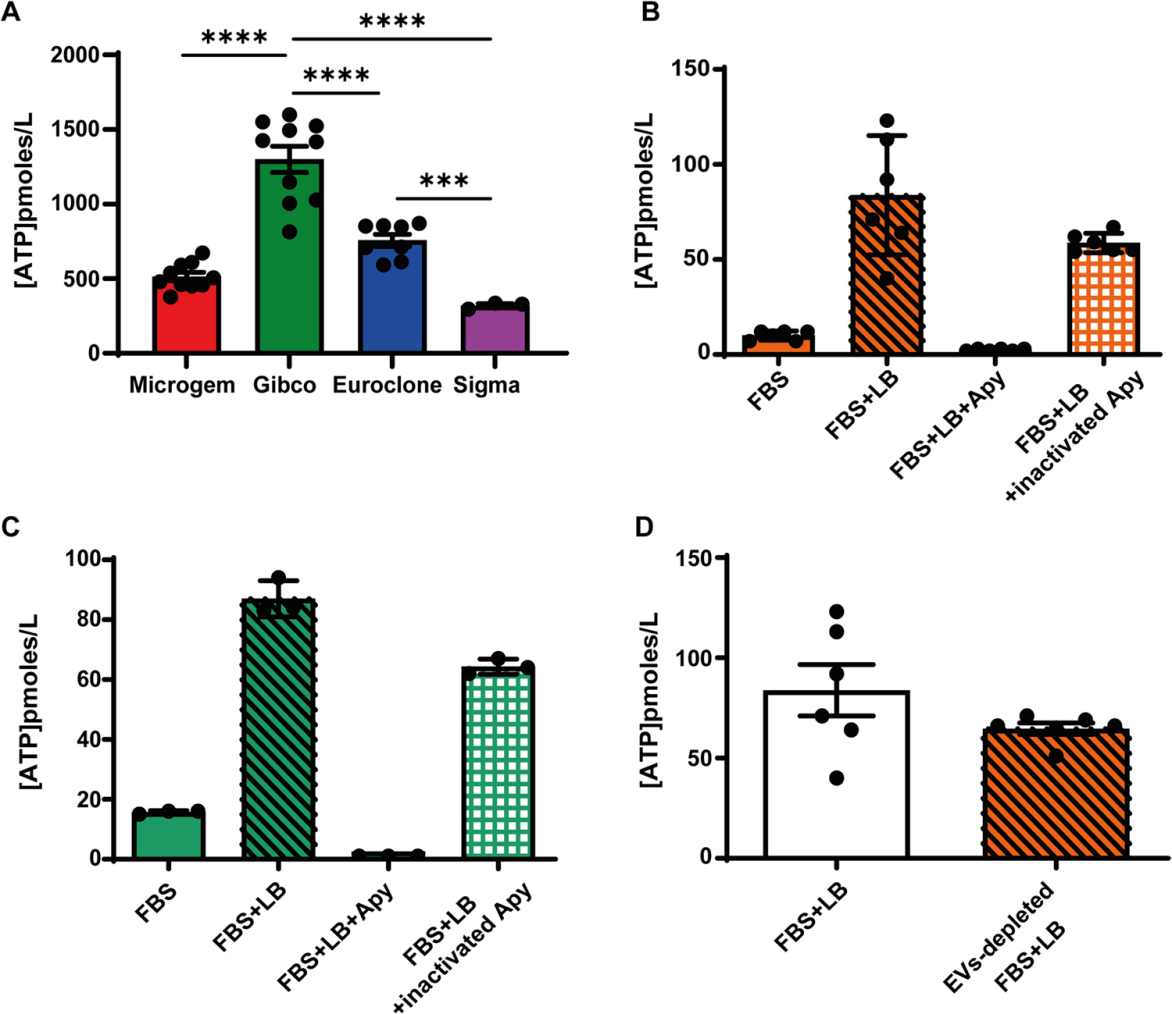
Fetal bovine serum (FBS) contains ATP and FBS-derived EVs/MPs are a source of ATP. **A** ATP concentration of four different commercially available FBS samples after ATP extraction using acid precipitation protocol. **B**-**C** ATP content of heat-inactivated (**B**) and non-inactivated (**C**) serum samples (Gibco). Fresh serum was treated with Triton X-100-based lysis buffer (LB), and then incubated with apyrase (Apy) or inactivated apyrase. **D** ATP content of fresh FBS sample and EV-depleted FBS after treatment with lysis buffer. ATP was measured using soluble luciferase in all experiments

ATP was extracted from four different commercially available FBS samples using the standard acid precipitation protocol routinely used to extract ATP from biological samples. Figure 1A shows that the ATP content of the various FBS samples varies widely from as little as 250 pmol/L (Sigma) to as much as 1300 pmol/L (Gibco). We then measured with the luciferase/luciferin assay the ATP content of a fresh serum (Gibco) sample, which yielded an ATP concentration about 100 times lower, i.e. about 18 pmol/L, suggesting that a large fraction of the ATP contained in FBS was not directly available for the luciferase assay (Fig. 1C). FBS contains a variable amount of blood-derived EVs/MPs known to be loaded with ATP [10]. Treatment with X-100-based detergent (lysis buffer, LB) increased fivefold the measurable ATP level, up to about 90 pmol/L. This shows that FBS contains a rather low amount of freely available ATP as well as an ATP reservoir that can be released after treatment with a detergent. Incubation of the samples with apyrase, a powerful soluble ATPase, abrogated the luciferase response, and accordingly apyrase inactivation prevented the apyrase-induced abrogation of the luciferase signal, proving the specificity of apyrase effect (Fig. 1C). As FBS for cell culture is routinely heat-inactivated at 56° C, we also tested the ATP content after heat inactivation (Fig. 1B), but there was no substantial difference versus non-inactivated serum (Fig. 1C). Release of ATP by treatment with lysis buffer suggested that FBS-contained EVs/MPs might be a source of ATP. In fact, FBS-derived EVs/MPs isolated by ultracentrifugation do indeed contain ATP to an amount of about 36 pmol/100 mL of serum (not shown). Thus, we measured the ATP content of FBS deprived of EVs/MPs by ultracentrifugation.

**Fig. 2:**
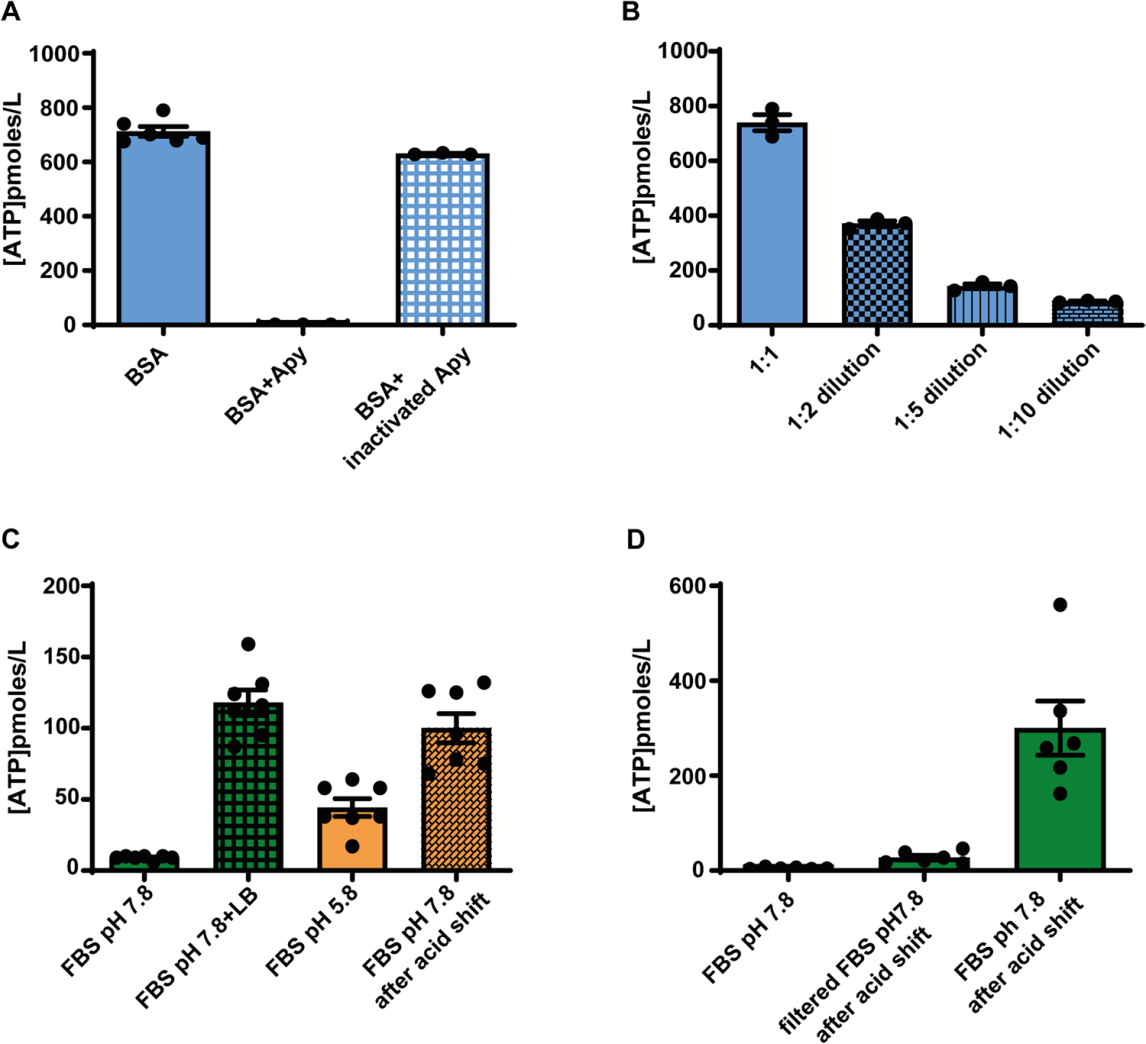
ATP binds to bovine serum albumin (BSA). **A** ATP content of a BSA solution (1 mg/mL) treated with triton X-100-based lysis buffer (LB) and incubated with either apyrase (Apy) or inactivated apyrase. **B** ATP concentration of a BSA solution at different dilutions. **C** ATP content of fresh FBS sample (Gibco) after treatment with LB at neutral pH (7.8) and after induction of an acid shock. ATP was measured at acid pH (5.8) and then at restored neutral pH. **D** ATP content of BSA-depleted FBS (filtered FBS) and complete (BSA-containing) FBS exposed to acid shock. ATP was measured using soluble luciferase in all experiments

As shown in Fig. 1D, removal of EVs/MPs caused only a small (about 20%) reduction of ATP content, indicating that the large majority of ATP was bound to a non-vesicular FBS fraction. Figure 1D shows that addition of lysis buffer to EV/MP-depleted FBS still triggered an increase in the free ATP concentration, although lower that complete FBS. Since lysis buffer is slightly acidic (pH 5.5), it is likely that the modest acidification caused by its addition releases ATP from an acid-sensitive non-vesicular reservoir. Previous reports show that ATP binds to BSA [11, 12] with a 1:1 stoichiometry, possibly at the fatty acid binding site [11]. We thus verified if commercial BSA binds ATP. An aqueous solution of BSA (1 mg/mL) treated with lysis buffer released an ATP amount of about 800 pmol/L, which was fully abrogated by apyrase, but not by inactivated apyrase (Fig. 2A). Free ATP was linearly related to the BSA concentration suggesting a direct binding to BSA (Fig. 2B). Enhanced ATP release from BSA by treatment with acidic lysis buffer suggests that an acidic shock may displace ATP from BSA. It was previously shown that the ATP dissociation constant from BSA is 13 µM at acid pH (5.4) and 120 µM at neutral pH (7.4) [12].

Since the isoelectric point of BSA is about 5.0–5.5 [14], it is likely that at neutral pH BSA “traps” ATP, which can be released at acid pH. Thus, FBS was exposed to a mild acid shock, causing a pH shift from 7.8 to about 5.8, and then restored a neutral pH optimum (7.4–7.8) for luciferase assay. The acidic shift released basically the same amount of ATP released by lysis buffer treatment (Fig. 2C). To verify whether ATP was bound to a high MW fraction, FBS was depleted of BSA and other high MW components by filtration through 50 K molecular-weight cut-off filters, and then both the BSA-depleted and BSA-enriched fractions were exposed to the acid shock and tested for ATP content. ATP was recovered only in the high MW, BSA-enriched, fraction (Fig. 2D), confirming that serum ATP is mainly bound to BSA or other high MW components.

**Fig. 3:**
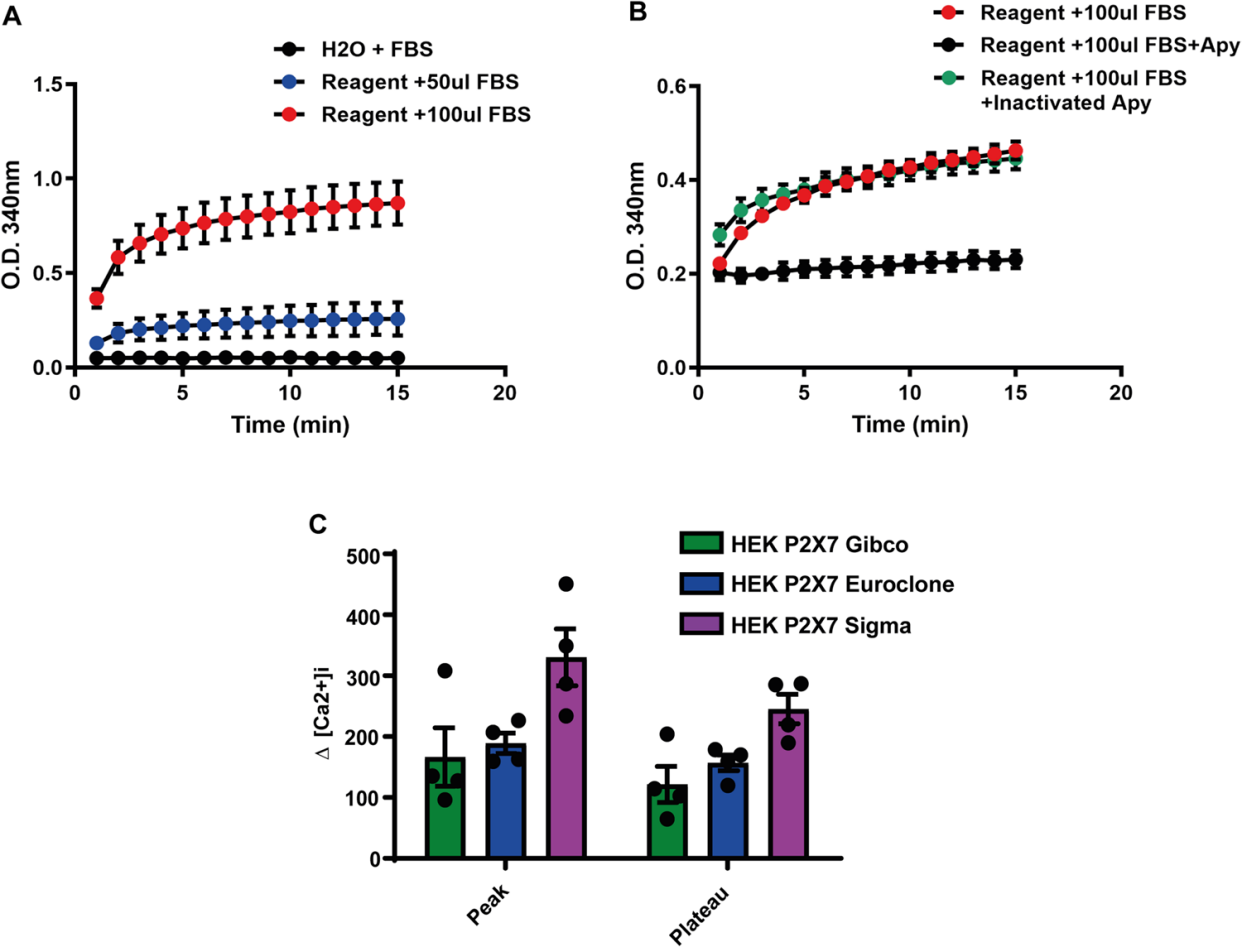
ATP in fetal bovine serum (FBS) is biologically available. **A** NADH absorbance increase at two different FBS concentrations. **B** NADH absorbance increase in FBS sample treated with apyrase (Apy) or inactivated apyrase. **C** [Ca^2+^]_i_ levels were measured with Fura 2/AM in HEK293-P2X7R cells (2 × 10.^6^) incubated overnight in DMEM-F12 medium supplemented with FBS from different commercial sources (Gibco, Euroclone, and Sigma), rinsed, resuspended in saline solution (see the “Materials and methods” section), and stimulated with ATP (1mM)

Then, we wondered if ATP contained in commercial FBS preparations is biologically available, i.e. can support biochemical reactions, such as for example glucose phosphorylation to generate glucose-6-phosphate. To this aim, FBS was added to a solution containing hexokinase and glucose-6-phosphate dehydrogenase (G6PDH) in the presence of NAD^+^ as co-factor. It is anticipated that, provided FBS contains ATP to a sufficient amount, glucose should be phosphorylated to glucose-6-phosphate in the reaction catalyzed by hexokinase. Glucose-6-phosphate is then oxidized to 6-phosphogluconate by G6PDH in the presence of NAD^+^, which is reduced to NADH. Thus, glucose-6-phosphate production, and therefore ATP usage, can be monitored by measuring the NADH absorbance increase. Figure 3A shows that FBS did indeed support glucose phosphorylation that was fully abrogated by apyrase, but not by inactivated apyrase (Fig. 3B).

Finally, we wondered whether such large variations in the ATP content of different FBS samples may affect cell responses. In principle, purinergic signalling might be modulated by the ATP content of culture medium as prolonged exposure to extracellular ATP may cause receptor desensitization. To verify this possibility, we measured the [Ca^2+^]_i_ of HEK293-P2X7R cells cultured in DMEM media supplemented with FBS from the different commercial sources, thus containing different ATP amounts. As shown in Fig. 3C, addition of exogenous ATP to HEK293-P2X7R cells incubated in culture medium supplemented with FBS with a lower ATP content (Sigma) triggered a higher [Ca^2+^]_i_ increase. Finally, we also investigated the FBS ATP content by mass spectrometry, but sensitivity of the assay was insufficient to reliably detect ATP in the serum samples (not shown).

## Discussion

Purinergic signalling is a ubiquitous intercellular communication system mainly based on release of ATP and its conversion to adenosine [15]. However, the extracellular environment is not completely free of purinergic agonists, as the extracellular ATP concentration in the interstitium of healthy tissues is thought to be in the low nanomolar range [16]. Therefore, extracellular ATP may affect cell responses either by keeping high affinity P2 receptors in a constitutive state of basal activation, or more likely by causing receptor desensitization. It is assumed that media routinely used for cell culture and in vitro experiments are basically ATP-free. This is of importance in the investigation of purinergic signalling because many P2Y and P2X receptors may desensitize after prolonged exposure to ATP [17, 18]. Our findings show that the ATP concentration in FBS, routinely used for cell cultures, is in the high picomolar/low nanomolar range, and in principle much lower at the final FBS dilution used in most in vitro experimental settings (1/10 FBS dilution). Yet, even at the very low concentration present in commercially available serum, ATP may support biochemical reactions, such as the phosphorylation of glucose to generate glucose 6-phosphate, and may also affect Ca^2+^ signalling by reducing the response to stimulation with exogenous ATP.

Since serum from different commercial sources contains different ATP amounts, investigation of purinergic signalling may be differentially affected by the use of sera containing different ATP concentrations. It had already been reported that serum components may affect purinergic signalling by lowering the potency of BzATP at the P2X7 receptor [13]. BSA replicated the FBS effect due to its binding to BzATP. ATP binding to BSA was previously shown and investigated [11, 12], thus supporting the conclusion that a large part of ATP carried by FBS is bound to albumin. However, our study shows that besides albumin, EVs/MPs are an additional source of ATP in serum. FBS as well as human serum carry a large amount of EVs/MPs released by endothelial cells, platelets, monocytes, or other circulating cells [4]. Parenchymal cells are also known to release EVs/MPs into the circulation [19]. A multiplicity of bioactive factors is carried by EVs/MPs, among which we recently also identified ATP [10].

Based on the present observations that prolonged exposure to even pico/nano molar extracellular ATP concentrations may desensitize P2 receptors, we may hypothesize that this may happen even more frequently in vivo at inflammatory sites, where extracellular ATP concentration is much higher than in healthy tissues. Moreover, sites of inflammation are characterized by low acid pH which can induce ATP release from albumin and extracellular microparticles, as we demonstrated in the present work.

Commercially available FBS undergoes a laborious processing during its way from the production to the research laboratories, thus it is surprising that a labile molecule such as ATP survives production, freezing, handling, shipping, and storage in the laboratories of destination. Our findings suggest that both the binding to albumin and sequestration within the EV/MP lumen stabilize ATP and prevent its hydrolysis. On the other hand, ATP in serum may be at least partially available for biochemical reactions as shown by its ability to support glucose phosphorylation to generate glucose-6-phosphate and to interact with P2Rs, potentially causing desensitization.

In conclusion, our findings show that commercial serum contains variable amounts of ATP that may affect biochemical reactions and cell responses.

## Data Availability

Data is contained within the article. Data not shown is available from the corresponding author upon reasonable request.

## References

[1] Jochems CE, van der Valk JB, Stafleu FR, Baumans V (2002). The use of fetal bovine serum: ethical or scientific problem?. Altern Lab Anim.

[2] Neubert E, Senger-Sander SN, Manzke VS, Busse J, Polo E, Scheidmann SEF, Schon MP, Kruss S, Erpenbeck L (2019). Serum and serum albumin inhibit in vitro formation of neutrophil extracellular traps (NETs). Front Immunol.

[3] Bernardini C, Algieri C, Mantia D, Zannoni A, Salaroli R, Trombetti F, Forni M, Pagliarani A, Nesci S (2021). Relationship between serum concentration, functional parameters and cell bioenergetics in IPEC-J2 cell line. Histochem Cell Biol.

[4] Lehrich BM, Liang Y, Fiandaca MS (2021). Foetal bovine serum influence on in vitro extracellular vesicle analyses. J Extracell Vesicles.

[5] Thery C, Witwer KW, Aikawa E, Alcaraz MJ, Anderson JD, Andriantsitohaina R (2018). Minimal information for studies of extracellular vesicles 2018 (MISEV2018): a position statement of the International Society for Extracellular Vesicles and update of the MISEV2014 guidelines. J Extracell Vesicles.

[6] Aswad H, Jalabert A, Rome S (2016). Depleting extracellular vesicles from fetal bovine serum alters proliferation and differentiation of skeletal muscle cells in vitro. BMC Biotechnol.

[7] Ochieng J, Pratap S, Khatua AK, Sakwe AM (2009). Anchorage-independent growth of breast carcinoma cells is mediated by serum exosomes. Exp Cell Res.

[8] Shelke GV, Lasser C, Gho YS, Lotvall J (2014) Importance of exosome depletion protocols to eliminate functional and RNA-containing extracellular vesicles from fetal bovine serum. J Extracell Vesicles 3:24783. 10.3402/jev.v3.24783 10.3402/jev.v3.24783 PMC4185091 25317276

[9] Zhou Q, Xie F, Zhou B, Li C, Kang Y, Wu B, Li L, Dai R (2020). Fetal bovine serum-derived exosomes regulate the adipogenic differentiation of human bone marrow mesenchymal stromal cells in a cross-species manner. Differentiation.

[10] Vultaggio-Poma V, Falzoni S, Chiozzi P, Sarti AC, Adinolfi E, Giuliani AL, Sanchez-Melgar A, Boldrini P, Zanoni M, Tesei A, Pinton P, Di Virgilio F (2022). Extracellular ATP is increased by release of ATP-loaded microparticles triggered by nutrient deprivation. Theranostics.

[11] Bauer M, Baumann J, Trommer WE (1992). ATP binding to bovine serum albumin. FEBS Lett.

[12] Takeda S, Miyauchi S, Nakayama H, Kamo N (1997). Adenosine 5′-triphosphate binding to bovine serum albumin. Biophys Chem.

[13] Michel AD, Xing M, Humphrey PP (2001). Serum constituents can affect 2′-& 3′-O-(4-benzoylbenzoyl)-ATP potency at P2X(7) receptors. Br J Pharmacol.

[14] Gaevskaia VA (1978). Azhitskii G (1978) [Isoelectric fractions of healthy human serum albumin and their ability to bind bilirubin]. Ukr Biokhim Zh.

[15] Burnstock G (2018). Purine and purinergic receptors. Brain Neurosci Adv.

[16] Giuliani AL, Sarti AC, Di Virgilio F (2019). Extracellular nucleotides and nucleosides as signalling molecules. Immunol Lett.

[17] Abbracchio MP, Burnstock G, Boeynaems JM, Barnard EA, Boyer JL, Kennedy C, Knight GE, Fumagalli M, Gachet C, Jacobson KA, Weisman GA (2006). International Union of Pharmacology LVIII: update on the P2Y G protein-coupled nucleotide receptors: from molecular mechanisms and pathophysiology to therapy. Pharmacol Rev.

[18] Illes P, Muller CE, Jacobson KA, Grutter T, Nicke A, Fountain SJ, Kennedy C, Schmalzing G, Jarvis MF, Stojilkovic SS, King BF, Di Virgilio F (2021). Update of P2X receptor properties and their pharmacology: IUPHAR Review 30. Br J Pharmacol.

[19] van der Pol E, Boing AN, Harrison P, Sturk A, Nieuwland R (2012). Classification, functions, and clinical relevance of extracellular vesicles. Pharmacol Rev.

